# Echocardiographic study of healthy Indian Spitz dogs with normal reference ranges for the breed

**DOI:** 10.14202/vetworld.2019.740-747

**Published:** 2019-06-01

**Authors:** Deepti Bodh, Mozammel Hoque, Abhishek Chandra Saxena

**Affiliations:** Division of Surgery, Indian Veterinary Research Institute, Izatnagar, Uttar Pradesh, India

**Keywords:** dogs, echocardiography, Indian Spitz, M-mode

## Abstract

**Aim:**

The present study was aimed to determine the normal reference values of M-mode echocardiographic measurements in healthy Indian Spitz dogs and evaluate the influence of gender and body weight on these measurements.

**Materials and Methods:**

M-mode echocardiography was performed in twenty-four clinically healthy conscious Indian Spitz dogs, aged 3-5 years and weighing 7-18 kg. Measurements were made from the right parasternal long axis left ventricular outflow tract view of the heart. The parameters recorded were: Left ventricular internal dimension, interventricular septal thickness and left ventricular posterior wall thickness during diastole and systole, left atrial diameter, aortic root diameter, left ventricular systolic functional parameters, and indices and mitral valve parameters.

**Results:**

M-mode echocardiographic measurements in healthy Indian Spitz dogs were standardized. Gender had no influence on echocardiographic measurements except mitral valve excursion amplitude and time interval between onset and end of mitral valve closure, which were significantly (p<0.05) higher in females than males. Left ventricular internal dimension at end-diastole, left ventricular internal dimension at end-systole, left ventricular posterior wall dimension at end-systole, end-diastolic volume, end-systolic volume, stroke volume, cardiac output, left ventricular ejection time, and mitral valve excursion amplitude correlated significantly (p<0.05) with body weight in Indian Spitz dogs.

**Conclusion:**

Data obtained in the present study can be used as breed-specific reference values for cardiac disease diagnosis as well as for future studies in Indian Spitz dogs.

## Introduction

Echocardiography is an important diagnostic tool used routinely for evaluating the structure and function of the heart as well as for diagnosing diseases of the heart. To determine cardiac performance in a healthy individual and diagnose pathological conditions in diseased animal, cardiac dimensions, cardiac functional parameters, and cardiac indices must be known.

Reference ranges of M-mode echocardiographic measurements for normal dog population including various breeds are published [1], but their clinical usefulness is limited due to the wide variation in the breed, body size, body weight, and body conformation among dogs [2,3]. Breed-specific reference echocardiographic values are published for various dog breeds such as Beagles [4], Bull Terriers [5], German shepherds [6], Whippets [7], Yorkshire Terriers [8], Indonesian Mongrel dogs [9], Border Collies [10], Labrador Retrievers [11,12], Rajapalayam dog [13], and Nigerian local dogs [14]. The Indian Spitz is a member of Spitz family that has received great interest as a companion pet in India. Echocardiographic measurements of Indian Spitz dogs might differ from other dog breeds due to the difference in its origin and predisposition to heart diseases [15-17]. Except for a few reports [18,19], detailed M-mode echocardiographic study in Indian Spitz dog lacks in the literature.

The present study aimed to determine the reference ranges of normal M-mode echocardiographic measurements in healthy Indian Spitz dogs and correlate them with gender and body weight of animals.

## Materials and Methods

### Ethical approval

Not necessary as the study was conducted on clinically normal conscious dogs that were presented for routine clinical examination. Written consent was obtained from dog owners before the start of the examination procedure.

### Animals

Twenty-four client owned, clinically healthy conscious Indian Spitz dogs (n=24; 12 males and 12 females) presented for routine health examination to the Referral Veterinary Polyclinics, Indian Veterinary Research Institute were included in this study. The mean age and body weight of dogs were 4.25±0.54 years (range=3-5 year) and 11.88±2.70 kg (range=7-18 kg), respectively. Dogs having normal cardiovascular and physical examination findings, without a history of cardiac disease and with normal results of six-lead electrocardiographic (ECG), as well as M-mode, two dimensional and Doppler echocardiographic examinations were selected.

### Echocardiography

Echocardiographic examination was performed with a Digital Color Doppler Ultrasound System (Chison iVis 60 Expert Vet^®^), Chison Medical Imaging Co., Ltd. equipped with color flow mapping and spectral Doppler mode, using a 3.5-5.5 MHz multi-frequency phased array transducer.

### Patient preparation and positioning

Right thoracic wall in the region between 3^rd^ and 7^th^ rib and adjoining 1-5 cm area lateral to sternum was prepared for the echocardiographic examination. The dogs were placed in the right lateral recumbency position on a table having a slit in the middle, enabling all examinations to take place from below the patient. The dogs were conscious throughout the examination, and no drugs were used.

### Measurements

M-mode dimensional measurements were obtained from the right parasternal long axis left ventricular outflow tract view of the heart. M-mode echocardiographic evaluation of heart was guided by the simultaneous display of real-time two-dimensional echocardiographic images. M-mode echocardiographic measurements were performed as per the guidelines of the American Society of Echocardiography using leading-edge to leading-edge method of measurement [20].

Left ventricular images were obtained by positioning the cursor at a point just posterior to the chordae tendineae perpendicular to interventricular septum and left ventricular posterior wall [3]. M-mode measurements recorded from the left ventricle included: Left ventricular internal dimension at end-diastole (LVIDd), left ventricular internal dimension at end-systole (LVIDs), left ventricular posterior wall dimension at end-diastole (LVPWd), left ventricular posterior wall dimension at end-systole (LVPWs), interventricular septal thickness at end-diastole (IVSd), and interventricular septal thickness at end-systole (IVSs).

Mitral valve measurements were made when opening and closing of the mitral valve leaflets was clearly visible. Various points on the mitral valve image in M-mode were identified and following measurements were recorded: 1) Mitral valve excursion amplitude (DE amplitude) which represents maximum excursion of cranial mitral valve leaflets during early diastolic filling of left ventricle, 2) Opening velocity of mitral valve during early diastole (DE slope), 3) Closing velocity of mitral valve during early diastole (EF slope), 4) Time interval between onset of mitral valve closure (A point) to end of mitral valve closure (C point) i.e. AC interval, 5) E-point to ventricular septal separation (EPSS) which represents the distance between interventricular septum and maximal initial opening of the mitral valve E-point.

Aortic root diameter (AOD) was measured at end-diastole from the top of the anterior aortic wall to the top of the posterior aortic wall. Measurements were made on a portion of the sweep with at least one visible valve cusp. Left atrial diameter (LAD) was measured at end-systole from the top of the posterior aortic wall to the top of the pericardium. LAD: AOD ratio was determined as an index to calculate the size of the left atrium.

### Calculations

Teichholz formula [21] was for the calculation of following indices:

End-diastolic volume (EDV) (ml) = 7 (LVIDd)^3^/2.4+LVIDd

End-systolic volume (ESV) (ml) = 7 (LVIDs)^3^/2.4+LVIDs

Stroke volume (SV) (ml/beat) = LVEDV-LVESV

Cardiac output (CO) (L/min) = (Heart rate×Stroke volume)/1000

Fractional shortening (FS) (%) = (LVIDd-LVIDs/LVIDd)×100

Ejection fraction (EF) (%) = (EDV-ESV/EDV)×100

Interventricular septum thickening fraction (IVSFT) (%) = (IVSs-IVSd)/IVSd×100

LV posterior wall thickening fraction (LVPWFT) (%) = (LVPWs-LVPWd)/LVPWd×100

Diastolic interventricular septum thickness to diastolic left ventricular posterior wall thickness ratio = IVSd/LVPWd

Diastolic interventricular septal thickness to diastolic left ventricular internal dimension ratio = IVSd/LVIDd

Diastolic left ventricular internal dimension to diastolic left ventricular posterior wall thickness ratio = LVIDd/LVPWd

Other indices were calculated using an established formula [5].

Velocity of circumferential fiber shortening (Vcf) (cm/s) = (LVIDd-LVIDs)/(LVIDd×LVET), with LVIDd and LVIDs in cm; LVET in second

LV myocardial fraction (LVfracd) = (IVSd+LVPWd)/(IVSd+LVPWd+LVIDd).

### Statistical analysis

Data are presented as mean values ± standard deviation (SD). Unpaired student’s t-test was used to determine differences between male and female dogs. Linear regression analysis was performed, and Pearson correlation coefficient (r) was calculated to determine the influence of body weight on echocardiographic parameters. The correlation was considered positive and significant when the correlation coefficient was ≥0.40, and significance was ≤0.05. Computer software SPSS version 17 (SPSS Inc., Chicago, IL) was used for statistical analysis. Value of p<0.05 and p<0.01 was considered significant at 5% and 1 %, respectively.

## Results

Mean ± SD of echocardiographic measurements in Indian Spitz dogs is summarized in Table-1. Mean ± SD of echocardiographic measurements in male and female Indian Spitz dogs is summarized in Tables-2 and 3, respectively. Body weight correlations of different echocardiographic measurements in Indian Spitz dogs are summarized in Table-4. Gender had no influence on echocardiographic measurements except DE amplitude and AC interval, which were significantly (p<0.05) higher in females than males. LVIDd, LVIDs, LVPWs, EDV, ESV, LVET, and DE amplitude demonstrated a positive correlation with body weight.

**Table-1:** M-mode echocardiographic measurements in Indian Spitz dogs.

Parameters (units)	Mean±SD (Total)	95% CI	Range
BW (kg)	11.88±2.70	10.43-13.32	7-18
LVIDd (mm)	35.15±4.77	32.61-37.69	28.80-46.50
LVIDs (mm)	22.54±3.65	20.59-24.48	18.80-29.30
IVSd (mm)	7.59±0.93	7.09-8.08	6.0-9.5
IVSs (mm)	13.25±2.47	11.94-14.56	8.2-17.30
LVPWd (mm)	8.19±1.07	7.62-8.76	6.20-10.00
LVPWs (mm)	12.21±1.38	11.48-12.95	10.10-14.80
LA (mm)	17.61±2.99	16.01-19.20	11.90-22.50
Ao (mm)	17.93±2.80	17.44-20.42	12.50-23.10
LA:Ao	0.93±0.09	0.89-0.98	0.74-1.08
EDV (ml)	53.85±18.29	44.10-63.60	31.67-99.65
ESV (ml)	17.53±7.07	13.76-21.29	10.89-32.98
SV (ml)	36.32±13.01	29.39-43.25	16.38-67.33
CO (L/min)	4.24±1.55	3.41-4.07	2.02-6.79
FS (%)	36.77±6.06	33.54-40.00	21.13-45.76
EF (%)	67.15±8.26	62.75-71.55	44.34-77.93
IVSFT (%)	74.12±20.30	63.30-84.94	18.84-111.68
LVPWFT (%)	49.77±11.16	43.82-55.72	25.58-62.90
IVSd/LVPWd	0.98±0.22	0.86-1.10	0.77-1.68
IVSd/LVIDd	0.22±0.04	0.20-0.24	0.17-0.29
LVIDd/LVPWd	4.32±0.54	3.30-5.48	4.03-4.61
LVET (s)	0.24±0.01	0.22-0.27	0.16-0.30
Vcf (circ/s)	1.44±0.39	1.23-1.65	0.64-2.08
LV fracd	0.31±0.03	0.29-0.33	0.26-0.37
DE amp. (mm)	12.13±2.65	10.71-13.54	6.0-17.0
DE slope (mm/s)	233.19±93.69	183.27-283.12	103.60-517.60
EF slope (mm/s)	119.84±25.21	106.41-133.28	78.60-152.40
AC interval (ms)	63.75±8.85	50-80	59.03-68.47
EPSS (mm)	4.62±1.15	4.01-5.24	3.0-7.0

p≤0.05, significantly different at 5%; CI=Confidence intervals, LVIDd=Left ventricular internal dimension at end-diastole, LVIDs=Left ventricular internal dimension at end-systole, IVSd=Interventricular septal thickness at end-diastole, IVSs=Interventricular septal thickness at end-systole, LVPWd=Left ventricular posterior wall thickness at end-diastole, LVPWs=Left ventricular posterior wall thickness at end-systole, LA=Left atrial diameter at end-systole, Ao=Aortic root diameter at end-diastole, LA:Ao=Left atrial to aortic root ratio, EDV=End-diastolic volume, ESV=End-systolic volume, SV=Stroke volume, CO=Cardiac output, FS=Fractional shortening, EF=Ejection fraction, IVSFT=Interventricular septal fractional thickening, LVPWFT=Left ventricular posterior wall fractional thickening, IVSd/LVPWd=Diastolic interventricular septum thickness to diastolic left ventricular posterior wall thickness ratio, IVSd/LVIDd=Diastolic interventricular septum thickness to diastolic left ventricular internal dimension ratio, LVIDd/LVPWd=Diastolic left ventricular internal dimension to diastolic left ventricular posterior wall thickness ratio, LVET=Left ventricular ejection time, Vcf=Velocity of circumferential fiber shortening, LV fracd=Left ventricular myocardial fraction, DE amplitude=Mitral valve excursion amplitude, DE slope=Mean velocity of early diastolic opening of mitral valve, EF slope=Mean velocity of mid diastolic partial closure, AC interval=Time interval between the peak of A wave and the C point, EPSS=E-point to ventricular septal separation, SD=Standard deviation

**Table-2:** M-mode echocardiographic measurements in male Indian Spitz dogs.

Parameters (units)	Mean±SD (Males)	95% CI	Range
BW (kg)	10.88±2.69	8.62-13.13	7-14
LVIDd (mm)	35.26±5.06	31.03-39.50	31-47
LVIDs (mm)	22.99±3.95	19.68-26.29	19-29
IVSd (mm)	7.34±1.15	6.37-8.30	6-10
IVSs (mm)	12.45±3.01	9.93-14.97	8-17
LVPWd (mm)	8.06±1.01	7.22-8.91	7-10
LVPWs (mm)	11.99±1.58	10.66-13.31	10-15
LA (mm)	16.89±3.55	13.92-19.86	12-22
Ao (mm)	17.81±2.92	15.37-20.25	13-21
LA:Ao	0.95±0.11	0.85-1.04	-
EDV (ml)	55.36±20.34	38.35-72.37	37-100
ESV (ml)	18.02±7.21	11.99-24.05	11-32
SV (ml)	37.34±15.32	24.53-50.15	16-67
CO (L/min)	3.76±1.42	2.58-4.94	2-6
FS (%)	36.59±7.76	30.10-43.08	21-46
EF (%)	66.60±10.69	57.67-75.53	44-78
IVSFT (%)	68.71±22.68	49.74-87.67	19-95
LVPWFT (%)	48.95±10.98	39.78-58.13	26-58
IVSd/LVPWd	0.91±0.10	0.83-1.00	-
IVSd/LVIDd	0.21±0.24	0.18-0.24	-
LVIDd/LVPWd	4.38±0.31	4.12-4.64	-
LVET (s)	0.26±0.03	0.23-0.28	-
Vcf (circ/s)	1.36±0.46	0.98-1.75	-
LV fracd	0.30±0.02	0.28-0.33	-
DE amp.(mm)	10.63±2.67	8.39-12.86	6-15
DE slope (mm/s)	193.40±65.24	138.86-247.94	104-287
EF slope (mm/s)	116.80±28.87	92.66-140.94	79-152
AC interval (ms)	58.75±6.41	53.39-64.11	50-70
EPSS (mm)	4.50±1.07	3.61-5.39	3-6

LVIDd=Left ventricular internal dimension at end-diastole, LVIDs=Left ventricular internal dimension at end-systole, IVSd=Interventricular septal thickness at end-diastole, IVSs=Interventricular septal thickness at end-systole, LVPWd=Left ventricular posterior wall thickness at end-diastole, LVPWs=Left ventricular posterior wall thickness at end-systole, LA=Left atrial diameter at end-systole, Ao=Aortic root diameter at end-diastole, LA:Ao=Left atrial to aortic root ratio, EDV=End-diastolic volume, ESV=End-systolic volume, SV=Stroke volume, CO=Cardiac output, FS=Fractional shortening, EF=Ejection fraction, IVSFT=Interventricular septal fractional thickening, LVPWFT=Left ventricular posterior wall fractional thickening, IVSd/LVPWd=Diastolic interventricular septum thickness to diastolic left ventricular posterior wall thickness ratio, IVSd/LVIDd=Diastolic interventricular septum thickness to diastolic left ventricular internal dimension ratio, LVIDd/LVPWd=Diastolic left ventricular internal dimension to diastolic left ventricular posterior wall thickness ratio, LVET=Left ventricular ejection time, Vcf=Velocity of circumferential fiber shortening, LV fracd=Left ventricular myocardial fraction, DE amplitude=Mitral valve excursion amplitude, DE slope=Mean velocity of early diastolic opening of mitral valve, EF slope=Mean velocity of mid diastolic partial closure, AC interval=Time interval between the peak of A wave and the C point, EPSS=E-point to ventricular septal separation, SD=Standard deviation, CI=Confidence intervals

**Table-3:** M-mode echocardiographic measurements in female Indian Spitz dogs.

Parameters (units)	Mean±SD (Females)	95% CI	Range
BW (kg)	12.88±0.88	10.81-14.94	10-18
LVIDd (mm)	35.04±4.79	31.03-39.05	29-42
LVIDs (mm)	22.09±3.53	19.14-25.04	19-29
IVSd (mm)	7.84±0.62	7.31-8.36	7-9
IVSs (mm)	14.05±1.58	12.73-15.37	13-17
LVPWd (mm)	8.33±1.17	7.34-9.31	6-10
LVPWs (mm)	12.44±1.20	11.43-13.44	10-14
LA (mm)	18.33±2.33	16.38-20.27	15-23
Ao (mm)	20.05±2.33	18.10-22.00	17-23
LA:Ao	0.91±0.05	0.87-0.96	-
IVSFT (%)	79.54±17.37	65.01-94.06	57-112
LVPWFT (%)	50.58±12.04	40.51-60.65	32-63
IVSd/LVPWd	1.05±0.29	0.81-1.29	1-2
IVSd/LVIDd	0.23±0.04	0.19-0.26	-
LVIDd/LVPWd	4.27±0.73	3.66-4.88	3-5
EDV (ml)	52.33±17.27	37.89-66.78	32-80
ESV (ml)	17.03±7.37	10.87-23.20	11-33
SV (ml)	35.30±11.19	25.99-44.66	20-50
CO (L/min)	4.72±1.63	3.36-6.08	3-7
FS (%)	36.95±4.29	33.37-40.54	31-43
EF (%)	67.70±5.60	63.01-72.38	59-75
LVET (s)	0.24±0.05	0.20-0.29	-
Vcf (circ/s)	1.52±0.34	1.24-1.80	-
LV fracd	0.32±0.04	0.29-0.35	-
DE amp.(mm)*	13.63±1.66	12.22-15.03	12-17
DE slope (mm/s)	268.00±63.26	185.58-360.40	189-518
EF slope (mm/s)	122.88±22.52	104.06-141.71	87-145
AC interval (ms)*	68.75±8.34	61.77-75.73	60-80
EPSS (mm)	4.75±1.28	3.68-5.82	3-7

LVIDd=Left ventricular internal dimension at end-diastole, LVIDs=Left ventricular internal dimension at end-systole, IVSd=Interventricular septal thickness at end-diastole, IVSs=Interventricular septal thickness at end-systole, LVPWd=Left ventricular posterior wall thickness at end-diastole, LVPWs=Left ventricular posterior wall thickness at end-systole, LA=Left atrial diameter at end-systole, Ao=Aortic root diameter at end-diastole, LA:Ao=Left atrial to aortic root ratio, EDV=End-diastolic volume, ESV=End-systolic volume, SV=Stroke volume, CO=Cardiac output, FS=Fractional shortening, EF=Ejection fraction, IVSFT=Interventricular septal fractional thickening, LVPWFT=Left ventricular posterior wall fractional thickening, IVSd/LVPWd=Diastolic interventricular septum thickness to diastolic left ventricular posterior wall thickness ratio, IVSd/LVIDd=Diastolic interventricular septum thickness to diastolic left ventricular internal dimension ratio, LVIDd/LVPWd=Diastolic left ventricular internal dimension to diastolic left ventricular posterior wall thickness ratio, LVET=Left ventricular ejection time, Vcf=Velocity of circumferential fiber shortening, LV fracd=Left ventricular myocardial fraction, DE amplitude=Mitral valve excursion amplitude, DE slope=Mean velocity of early diastolic opening of mitral valve, EF slope=Mean velocity of mid diastolic partial closure, AC interval=Time interval between the peak of A wave and the C point, EPSS=E-point to ventricular septal separation, SD=Standard deviation, CI=Confidence intervals

**Table-4:** Body weight correlation with echocardiographic measurements in Indian Spitz dogs.

Parameters (unit)	Regression (y=)	Coefficient of determination (r^2^)	Correlation (r)	p-value for correlation
LVIDd (mm)	1.143x+21.58	0.420	0.648	0.003**
LVIDs (mm)	0.707x+14.13	0.275	0.524	0.018*
IVSd (mm)	0.086x+6.562	0.062	0.2505	0.1747
IVSs (mm)	0.3362x+9.257	0.1359	0.3686	0.0800
LVPWd (mm)	0.1448x+6.475	0.1341	0.3662	0.0815
LVPWs (mm)	0.227x+9.516	0.198	0.445	0.041*
LAD (mm)	0.3828x+13.06	0.1195	0.3457	0.0948
AOD (mm)	0.3113x+15.23	0.090	0.3006	0.1289
EDV (ml)	4.600x−0.7765	0.462	0.680	0.002**
ESV (ml)	1.433x+0.5152	0.301	0.548	0.014**
SV (ml)	3.167x−1.287	0.433	0.659	0.0028**
CO (L/min)	0.2468x+1.310	0.184	0.429	0.048**
FS (%)	0.5567x+30.16	0.061	0.2485	0.1767
EF (%)	0.7396x+58.37	0.058	0.2421	0.1831
DE amp. (mm)	0.439x+6.904	0.2006	0.447	0.041*
DE slope (mm/s)	8.146x+136.5	0.055	0.2352	0.1903
EF slope (mm/s)	2.544x+89.69	0.074	0.2729	0.1532
EPSS (mm)	−0.1526x+6.437	0.1294	−0.3598	0.0855
LVET (s)	0.0067x+0.1685	0.1655	0.4068	0.0589*
Vcf (cir/s)	−0.020x+1.680	0.018	0.1376	0.3056

y is the achieved value of echocardiographic parameter at body weight x.

* S=Significant at p<0.05;

** S=Significant at p<0.01. r>0.40 and p<0.05 represent positive and significant correlation. LVIDd=Left ventricular internal dimension at end-diastole, LVIDs=Left ventricular internal diameter at end-systole, IVSd=Interventricular septal thickness at end-diastole, IVSs=Interventricular septal thickness at end-systole, LVPWd=Left ventricular posterior wall thickness at end-diastole, LVPWs=Left ventricular posterior wall thickness at end-systole, LAD=Left atrial diameter, AOD=Aortic root diameter, EDV=End-diastolic volume, ESV=End-systolic volume, SV=Stroke volume, CO=Cardiac output, FS=Fractional shortening, EF=Ejection fraction, DE amplitude=Mitral valve excursion amplitude, DE slope=Mean velocity of early diastolic opening of mitral valve, EF slope=Mean velocity of mid-diastolic partial closure, EPSS=E-point to ventricular septal separation, LVET=Left ventricular ejection time, Vcf=Velocity of circumferential fiber shortening

## Discussion

As limited information is available in the field of echocardiographic study in Indian Spitz dogs, the present study was designed to determine the reference ranges of normal M-mode echocardiographic measurements in healthy Indian Spitz dogs of both the sexes and study the possible effect of gender and body weight on echocardiographic measurements.

Left ventricular internal dimension, interventricular septal, and left ventricle posterior wall thickness in Indian Spitz dogs were in accordance with the range of values considered normal for dogs having similar body size [1]. LVIDd and LVIDs in Indian Spitz were greater than the previously reported value in Indian Spitz (29.37±0.64 mm and 19.71±0.88 mm) [18] but similar to Indian dogs (34.60±0.81 mm and 22.00±0.58 mm) [22]. LVIDd and LVIDs were unaffected by gender. Similar findings were reported by Boon [1], Kayar *et al*. [6], Gugjoo *et al*. [11], and Saxena [18]. However, in Whippets, Bavegems *et al*. [7] reported significantly larger values of left ventricular internal diameter in females, which was attributed to greater mean heart weight to body weight ratio of females as compared to males. Body weight correlated significantly (p<0.05) with diastolic and systolic left ventricular internal dimensions in Indian Spitz, similar to findings reported in previous studies [1,6,8,11,14,22], while in Pembroke Welsh Corgis and Afghan hounds, LVIDd was reported to be correlated non-significantly with body weight [2]. Alteration in the left ventricular internal diameters during diastole and systole is suggestive of either dilated or hypertrophic cardiomyopathy.

IVSd in Indian Spitz was similar to the previously reported value in Indian Spitz (7.83±1.44 mm) [18] but slightly lower than Indian dogs (9.70±0.28 mm) [22]. IVSs in Indian Spitz was similar to Indian dogs (14.1±0.40 mm) [22] but slightly lower than the previously reported value in Indian Spitz (12.33±1.49 mm) [18]. The non-significant effect of gender on diastolic and systolic IVS thickness in the present study was in line with the findings of other researchers [6,11,18]. Body weight did not correlate significantly with IVS dimensions in Indian Spitz, similar to findings in Beagle [4] and Labrador retriever dogs [12], while in Nigerian Mongrel dogs body weight was found to be correlated significantly with IVSd and IVSs [14].

LVPWd and LVPWs in Indian Spitz were slightly lower than the previously reported value in Indian Spitz (9.27±0.19 mm and 14.53±0.46 mm) [18] but similar to Indian dogs (8.9±0.26 mm and 11.8±0.34 mm) [22]. Left ventricular posterior wall dimensions in Indian Spitz did not correlate significantly with gender. In contrast, in other investigations, higher values of LVPWd and LVPWs in Beagle [4] and German shepherd dogs [6] were reported in males compared to females. Body weight did not correlate significantly with LVPWd in Indian Spitz, similar to findings in Miniature poodles, Afghan hounds [2], Greyhounds, Whippets, Italian Greyhounds [3], and Labrador retriever dogs [12]. However, body weight was found to be correlated significantly (p<0.05) with LVPWs, similar to findings of Boon [1]. In Nigerian Mongrel dogs, body weight was found to be correlated significantly with LVPWd and LVPWs [14] while other researchers [2,12] did not report any significant correlation between body weight and left ventricular posterior wall dimensions.

LAD was similar to the previously reported value in Indian Spitz (18.00±0.70 mm) [18] while AOD was slightly larger than the previously reported value in Indian Spitz (16.50±1.03 mm) [18]. However, both LAD and AOD in Indian Spitz were lower than Indian dogs (23.8±0.50 mm and 21.7±0.46 mm) [22]. Gender had no influence on LAD and AOD in Indian Spitz dogs. In contrast, in Indonesian Mongrel dogs, AOD was found to be larger in males while LAD and LAD/AOD ratios were found to be larger in females [9]. Body weight did not correlate significantly with LAD and AOD in Indian Spitz, similar to findings in German shepherd dogs [6]. While in Labrador retriever dogs, only AOD was reported to be correlated significantly to body weight [12]. Contrary to our findings, many researchers documented a significant positive correlation between body weight and left atrial and aortic root dimensions in various dog breeds [1,6,22]. Normal left atrial to aortic root ratio (LAD: AOD) in healthy dog lies between 0.8 and 1.2 [1]. LAD: AOD ratio in Indian Spitz was within this range and similar to previously reported value in Indian Spitz (0.98±0.01) [18] but lower than Bull Terriers (1.7±0.2 mm) [5], Yorkshire Terriers (1.37±0.12 mm) [8], Border Collies (1.16±0.21 mm) [10], and Indian dogs (1.10±0.15) [22]. Increased LAD may lead to greater left atrial to aortic root ratio, frequently seen in aged animals because the diameter of left atrium increases while that of aortic root decreases with advancing age.

End-diastolic volume, end-systolic volume, and stroke volume in Indian Spitz were greater than the previously reported values in Indian Spitz (33.24±1.04 ml; 12.44±1.38 ml; and 20.79±1.49 ml/beat) [18] but similar to Indian dogs (53.79±27.56 ml; 18.19±11.26 ml; and 35.58±18.53 ml/beat) [22]. The statistically non-significant difference in left ventricular volumes between male and female dogs was in accordance with the findings of other researchers [11,18]. Body weight correlated significantly (p<0.01) with left ventricular volumes, similar to findings reported in previous studies [11,12]. Cardiac output was greater than the previously reported value in Indian Spitz (2.133±0.19 L/min) [18]. Higher cardiac output and stroke volume in males compared to females were in accordance with findings in Whippets, where male Whippets had significantly higher cardiac output and stroke volume than female Whippets [7]. Cardiac output is a measure of global cardiac function but is not a sensitive indicator of cardiac performance because it is affected by many compensatory mechanisms such as heart rate and stroke volume which act to maintain a normal cardiac output.

FS and EF in Indian Spitz were similar to Indian dogs (36.61±6.98% and 66.81±9.15%) [22] but slightly greater than the previously reported value in Indian Spitz (32.69±2.86% and 62.57±4.07%) [18]. Our results regarding the lack of correlation between body weight and FS and EF was in agreement with the findings of other researchers [1,12,18], suggesting that these measurements are independent of body weight. However, other investigators reported a weak negative correlation between body weight and FS and EF [6,11]. Variation in the observation of FS and EF might result due to the dependence of these parameters on multiple factors such as preload, afterload, and contractility, which influence them [3,5].

Degree of thickening of the interventricular septum and left ventricle posterior wall, respectively, during ventricular contraction is indicated by IVSFT and LVPWFT. IVSFT was greater than Yorkshire Terriers (47.17%) [8], Indian Spitz (57.02±2.88%) [18], and healthy Indian dogs (49.29±28.07%) [22]. LVPWFT was greater than Yorkshire Terriers (47.17%) [8] and Indian dogs (34.45±25.44%) [22] but lower than the previously reported value in Indian Spitz (56.79±2.99%) [18]. Reduced percent change in the thickness of interventricular septum and posterior wall of the left ventricle during the cardiac cycle indicates reduced contractility of the myocardium, which might be attributed to long-standing volume overload. IVS and LVPW thickening fraction showed no significant variation with respect to gender. A similar finding was reported in previous studies [12,18]. No relationship between LVPW thickening fraction and body size measurements in Greyhounds, Whippets, and Italian Greyhounds suggested that this functional index is less affected by the wide variation in canine body sizes [3]. Ratios of IVSd/LVIDd and IVSd/LVPWd are used to rule out asymmetric septal hypertrophy or assess the extent of compensatory hypertrophy resulting from left ventricular outflow tract obstruction. LVIDd/LVPWd ratio assesses the degree of compensatory hypertrophy during cardiac disease process [1]. A normal LVIDd/LVPWd ratio along with volume overload of left ventricle suggests appropriate compensatory hypertrophy. Increase in LVIDd/LVPWd ratio indicates inadequate hypertrophy as in dilated cardiomyopathy while a decrease in LVIDd/LVPWd ratio indicates excessive hypertrophy as in hypertrophic cardiomyopathy.

Left ventricular ejection time (ET) in Indian Spitz was greater than Greyhounds (0.17 s), Whippets (0.18 s) and Italian Greyhounds (0.15 s) [3], and Whippets (0.16 s) [7] and Yorkshire Terriers (0.18±0.02 s) [8]. Vcf represents variation in the circumference of the ventricular cavity during systole. Vcf in Indian Spitz was lower than normal dogs (2.2±0.40 circ/s) [1], Whippets (1.69±0.39 circ/s) [7], and Yorkshire Terriers (2.42±0.56 circ/s) [8]. Ejection time is affected by the volume of blood within the left ventricle. With a reduction in preload decrease in ET occurs and vice versa. With an increase in afterload, ET increases and Vcf decreases. However, an increase in both ET and Vcf is seen when afterload decreases [1]. Left ventricular myocardial fraction (LV fracd) is also an indicator of hypertrophy. LV fracd in Indian Spitz was slightly lower than Indian dogs (0.35±0.06) [22]. The non-significant difference in the values of ET, Vcf, and LVfrac was observed between male and female Spitz dogs. In contrast, left ventricular ET was found to be significantly higher, and Vcf was found to be significantly lower in female whippets than males [7].

Mitral valve DE amplitude in Indian Spitz was similar to the previously reported value in Indian Spitz (11.67±0.15 mm) [18]. DE amplitude was found to be significantly (p<0.05) higher in females than males. This might be due to the higher average body weight of females resulting in higher ventricular diameters than males. Contrary to this, Saxena [18] reported significantly (p<0.05) higher DE amplitude in male Spitz, which could be due to more body weight of male Spitz used in their study. Body weight correlated significantly (p<0.05) with DE amplitude in accordance with findings in German shepherd [6], Labrador retriever [11], and Indian Spitz dogs. [18]. Diastolic excursion of a cranial mitral valve leaflet is flow related. With a decrease in systolic outflow as in dilated cardiomyopathy, diastolic inflow decreases resulting in decreased excursion of the cranial mitral valve leaflet.

Mitral DE slope in Indian Spitz was 233.19±93.69 mm/s. Body weight did not correlate significantly with DE slope in Indian Spitz, contrary to findings in German shepherd dogs [6]. Mitral EF slope represents the rate of left ventricular filling or left atrial emptying and is, therefore, an important indicator of flow across the mitral valve and mitral valve function. EF slope in Indian Spitz (119.84±25.21 mm/s) was similar to previously reported values in Indian Spitz (118.13±2.78 mm/s) [18]. We did not find any association between body weight and mitral EF slope. Contrary, Kayar *et al*. [6], Gugjoo *et al*. [11], and Saxena [18] reported a significant correlation between body weight and EF slope in German shepherd dogs, Indian Spitz, and Labrador retriever dogs, respectively. The non-significant effect of gender on mitral DE slope and EF slope in Indian Spitz was in accordance with findings in German shepherd dogs [6]. The time interval between onset of mitral valve closure to end of mitral valve closure, i.e. AC interval in Indian Spitz was 63.75±8.85 ms. Males had significantly (p<0.05) higher AC interval compared to females. Prolongation of AC interval is suggestive of elevated left ventricular end-diastolic pressure and poor ventricular function. Mitral valve EPSS was lower than the previously reported value in Indian Spitz (6.07±0.66 mm) [18] but greater than Indian dogs (3.4±0.12) [21]. The non-significant effect of gender on EPSS was observed in the present study. Contrary to our findings, Bavegems *et al*. [7] reported significantly higher EPSS in female whippets than males while Noviana *et al*. [9] reported significantly higher EPSS in male Indonesian Mongrel dogs than females. Some researchers documented a significant influence of body weight on EPSS [3,10,12], while others suggested a non-significant influence of body weight on the value of this parameter [6,7,22]. In the present study, body weight did not have a significant influence on EPSS.

## Conclusion

It can be concluded that the reference ranges of M-mode echocardiographic measurements in healthy Indian Spitz dogs were standardized. A significant influence of body weight and a non-significant influence of gender on the majority of M-mode echocardiographic parameters in Indian Spitz dogs were reported. Values obtained in the present study can be used as a reference for determination of cardiac function as well as for diagnosis of cardiovascular disease in Indian Spitz dogs.

## Authors’ Contributions

The present study is a part of DB’s Ph.D. dissertation. The work was designed by MH and ACS. Echocardiographic examination was performed by DB and ACS. Statistical analysis and drafting of the manuscript were done by DB. All authors read and approved the final manuscript.
